# Secular Improvements in Cognitive Aging: Contribution of Education, Health, and Routine Activities

**DOI:** 10.1177/08982643211065571

**Published:** 2022-01-12

**Authors:** Johannes Beller, Beatrice G. Kuhlmann, Stefanie Sperlich, Siegfried Geyer

**Affiliations:** 1 Medical Sociology Unit, 9177 Hannover Medical School, Hannover, Germany; 2 Cognitive Psychology and Cognitive Aging, University of Mannheim, Mannheim, Germany

**Keywords:** cognitive functioning, cognitive performance, aging, morbidity, trends

## Abstract

**Objectives:**

Limited evidence exists regarding the reasons for secular changes in cognitive functioning over historical time. Thus, we examined potential explanatory factors for changes in cognitive speed, a central dimension of cognitive functioning.

**Methods:**

Population-based data of middle-aged and older adults from Germany (*N* = 5443) was used with baseline participants from 2002 to 2014, comparing the time periods 2002–2014.

**Results:**

Cognitive speed improved in middle-aged adults (40–65) and older adults (66+). In both age groups, increases were partly explained by education, employment status, volunteering status, routine activities, and physical functioning. Changes in education were more important in explaining increases in older than in middle-aged adults, whereas changes in health were more important for explaining increases in middle-aged adults.

**Conclusions:**

Cognitive speed increased in both age groups over historical time. Education, employment, volunteering, routine activities, and health were all important in explaining these changes, but their importance differed between age groups.

Cognitive functioning—a person’s mental abilities—represents an important aspect of overall health and is one of the many health-related aspects declining with increasing adult age. In population-based studies, cognitive functioning has been associated with well-being, limitations in daily functioning, overall morbidity, and mortality risk (Langa et al., 2008). Cognitive functioning itself, on the other hand, has been found to be determined by a multitude of factors: Early education, occupation, health status, and later-life regular activities like visiting friends have been found to be associated with cognitive functioning (Grasshoff et al., 2021; Kelly et al., 2017; Llewellyn et al., 2008; Menec, 2003; Walsemann & Ailshire, 2020). Hence, cognitive functioning represents a key prerequisite of human health and it is vital for successful aging.

Several studies have analyzed inter-individual trends or changes in cognitive functioning over time, whether cognitive functioning has improved, remained stable, or declined over time in the general population (e.g., Henchoz et al., 2020; Hessel et al., 2018; Weuve et al., 2018). In analyses like these, one is interested in the between-person changes in cognitive functioning over historical time in the general population, as opposed to the within-person changes in cognitive functioning across age. If levels of cognitive functioning have increased and will continue to increase, future older adults may be able to maintain their overall health and daily functioning for a longer time—a scenario which has been described as a compression of morbidity (Fries, 1980; Fries et al., 2011; Langa et al., 2008). Contrarily, if cognitive functioning is decreasing in the face of increasing life expectancy, overall health and daily functioning of future cohorts of older adults might decrease—a scenario that was described as expansion of morbidity (Gruenberg, 1977; Kramer, 1980). Evidence for increasing levels of cognitive functioning has been reported in the literature, with few exceptions (Hale et al., 2020; Trahan et al., 2014).

However, to date, only some studies have empirically examined potential reasons for these historical trends in cognitive functioning. Studies that have analyzed potential explanations, were mostly focused on the role of school education. Weuve and colleagues found that improvements in cognitive functioning were accompanied largely by increasing educational levels in later cohorts (Weuve et al., 2018). The authors assumed that increasing levels of education might have led to higher levels of cognitive functioning. In another study, it was investigated whether trends in cognitive functioning might be explained by health status in addition to early education (Hessel et al., 2018). It was found that both partially explained increases in cognitive functioning over time. However, to our knowledge, no studies to date have examined how the overall life situation of older adults—including their education, health status, occupational status, and regular activities—might explain trends in cognitive functioning in a multivariate design. This will be examined in the current investigation: We analyze how cognitive speed has changed over historical time in the general population by comparing two population-based samples from different time point, and potentially important explanatory factors of these historical changes in cognitive speed between samples are examined. We ask: How can secular trends in middle-aged and older adults’ cognitive speed, a central dimension of cognitive functioning, be explained?

## Methods

### Sample

We used baseline data from the public release of the 2002 and the 2014 wave of the German Aging Survey (Deutscher Alterssurvey; DEAS), a cohort-sequential longitudinal, population-based study on Germans aged 40 years and older, provided by the Research Data Center of the German Center of Gerontology (Klaus et al., 2017; Mahne et al., 2020; Motel-Klingebiel et al., 2016). For the German Aging Survey, participants were drawn randomly by probability sampling in 2002 and 2014 and interviewed face-to-face in their residence. We used data from all baseline participants in 2002 and all baseline participants in 2014 who filled out a drop-off questionnaire, resulting in a sample size of *N* = 7136. After deleting missing values (about 24%) listwise, a final sample size of *N* = 5443 resulted (*N*_2002_ = 1874; *N*_2014_ = 3569). The degree to which cognitive speed has changed over time in the general population can be estimated by comparing the average cognitive speed between these two population-based samples, as the difference in cognitive speed between those two samples corresponds to the changes in cognitive speed over historical time. The sample was split according to age in a sample of middle-aged adults (40–65 years old) and older adults (66+ years old). However, as can be seen in the Appendix Table A1, our main findings are replicated even after imputing missing values by means of the non-parametric missForest algorithm, that has been developed for imputing mixed-type data where traditional assumptions like normality cannot be ascertained (Stekhoven & Buhlmann, 2012). Additionally, as can be seen in the Appendix Table A2, our main results are also replicated when applying weights provided by the German Aging Survey, with which the sample can be adjusted to mirror the German population in certain socio-demographic characteristics (Klaus et al., 2017). All participants provided informed consent.

### Measures

The German version of the Digit Symbol Substitution Test, obtained via a face-to-face interview, was used as the dependent variable. This test was derived from the Wechsler Intelligence Test. In the paper-and-pencil test, the respondents are presented with a table containing the Arabic numerals 1 to 9, each of which is assigned a simple geometric character. Participants then have 90 seconds to insert the appropriate character on a sheet of paper with rows of digits one after the other. Thus, the participant must match symbols to numbers using a reference at the top of the page. Theoretically the scores can range from 0 (no symbol is matched correctly to the Arabic numerals) to 93, the maximum number of provided Arabic numerals (all symbols are matched correctly to the Arabic numerals). However, it is generally not possible to match all characters correctly, since the Digit Symbol Substitution Test is a speed test, and the average correctly matched characters are thus typically much lower. The total number of correctly filled in characters was used as the indicator of cognitive speed. The Digit Symbol Substitution Test is one of the most commonly used tests in neuropsychology. It has been found to cover a wide range of cognitive abilities including visual-motor coordination, attention, associative learning, and working memory (Jaeger, 2018). The test has been employed in survey research on cognitive abilities and is well validated for this use (Hoyer et al., 2004). In cognitive aging research, this well-established measure of basic cognitive processing speed has been shown to explain a large proportion of the variance in older adults’ performance in a range of higher-order cognitive functions (Salthouse, 1996). Several indicators that were assessed in the 2002 and the 2014 wave were used to measure possible explanatory variables for inter-individual trends in cognitive speed: Educational level, employment status, volunteering status (whether the person executes an honorary office in the groups or organizations in which he is a member), computer use frequency, visiting friends frequency, brain sport frequency, sports frequency, game playing frequency, taking courses frequency and physical functioning. Educational level was coded taking both school education and professional training/academic training into account, resulting in a four-level classification scheme: [1] Participants without completed vocational qualification and up to a maximum of a graduation degree, [2] participants with vocational qualifications or qualifications for university entrance, [3] participants with finished upgrading training (e.g., as a master craftsman), and [4] participants with completed university studies. Employment status was operationalized by whether participants were currently working [1] or not working [0]. Computer use frequency, visiting friends frequency, brain game frequency, physical exercise frequency, game playing frequency, and taking courses frequency were operationalized by inquiring about the frequency with which one has done these respective activities within the last year ([1] = “never,” [2] = “less than monthly,” [3] = “1 to 3 times a monthly,” [4] = “weekly,” [5] = “multiple times per week,” [6] = “daily”). Physical functioning was measured with the subscale Physical Functioning of the German version of the Short Form 36 Health Survey (Bullinger, 1995), with scores raging theoretically from 100 (best physical functioning) to 0 (worst physical functioning). Additional covariates included time period ([0] = “2002” vs. [1] = “2014”), age (in years) and gender ([0] = “male” vs. [1] = “female”).

### Data Analysis

Mean comparisons and Spearman correlation analyses were calculated to provide basic descriptive statistics of inter-individual changes between the 2002 and 2014 samples. Additionally, multiple linear regression analyses were used to examine the degree to which cognitive speed changed over time in middle-aged and older adults inter-individually from 2002 to 2014 and whether these changes could be explained by our predictor variables. For this purpose, five models with increasing complexity were calculated based on theoretical considerations: The first model only included time period, age, and gender. The second model also included education to examine how the effect attributable to the time period changes once differences in educational status between survey waves are controlled for. The third model additionally includes employment status and volunteering status. The fourth model includes the six variables relating to frequency of routine activities: Computer Use, Visiting Friends, Brain Games, Doing Sports, Playing Games, and Taking Courses. Lastly, the fifth model also includes Physical Functioning. Changes in the effect size of the regression coefficient for time period between models indicate that the changes in cognitive speed across time might be attributed to the factors added in the respective model (MacKinnon, 2000). As such, the main predictor of interest “time period” constitutes the average difference in cognitive speed between the 2002 sample and the 2014 sample, controlled for a varying number of covariates. By comparing the size of the effect of “time period” (the average difference in cognitive speed between the 2002 sample and the 2014 sample) between different models with varying cofounders controlled for, we can estimate the degree to which these confounders explain the observed historical time period differences.

## Results

Descriptive statistics are reported in Table 1. The group of middle-aged participants (40–65 years) were on average 52.70 years old in 2002, and in 2014, middle-aged participants were on average 53.99 years old. Cognitive speed increased from 2002 (M = 45.83) to 2014 (M = 49.16) in middle-aged adults, with a small-to-moderate effect size of Cohen’s *d* = 0.25. The group of older participants (66+ years) were on average 73.96 years old in 2002, and in 2014, older participants were on average 73.49 years old. In this old age group, cognitive speed increased from 2002 (M = 33.94) to 2014 (M = 37.21) with a small-to-moderate effect size of Cohen’s *d* = 0.26. In 2002, about 4.6% of middle-aged participants had comparatively “low” cognitive speed scores more than 1.5 standard deviations below the overall sample average, whereas in 2014 only 2.5% scored more than 1.5 standard deviations below the overall sample average. Regarding older adults, in 2002, about 18.7% had comparatively “low” cognitive speed scores more than 1.5 standard deviations below the overall sample average, whereas in 2014 only 10.1% scored more than 1.5 standard deviations below the overall sample average. The intercorrelations between our variables are displayed in Table 2. All explanatory variables correlated significantly with cognitive speed in middle-aged adults, with age, employment, and computer use having the strongest associations. In older adults, all explanatory variables except gender and employment correlated significantly with cognitive speed, with age, computer use and education having the strongest associations.

**Table 1.** Descriptive Statistics of our Variables between 2002 and 2014 in Middle-Aged and Older Adults (N = 5443).

	Middle-Aged Adults (N = 3330)				Older Adults (N = 2113)			
	2002 (*N* = 1172)		2014 (*N* = 2158)		2002 (*N* = 702)		2014 (*N* = 1411)	
	M	SD	M	SD	M	SD	M	SD
Cognitive speed (DSST)	45.83	13.69	49.16	12.79	33.94	13.21	37.21	11.8
Age	52.7	7.46	53.99	7.06	73.96	4.72	73.49	4.78
Gender (female %)	51%	—	54%	—	48%	—	45%	—
Education (%)								
Low	7%	—	4%	—	23%	—	10%	—
Intermediate	56%	—	54%	—	52%	—	50%	—
Upper-intermediate	13%	—	15%	—	11%	—	15%	—
High	23%	—	28%	—	14%	—	25%	—
Working (%)	62%	—	70%	—	0%	—	0%	—
Volunteering (%)	22%	—	29%	—	14%	—	25%	—
Computer use	2.70	1.94	4.60	1.81	1.41	1.27	3.12	2.24
Visiting friends	3.20	0.96	3.18	0.94	2.94	1.09	2.97	0.96
Brain games	2.96	1.79	3.01	1.82	3.40	1.99	3.71	2.10
Sports	3.07	1.70	3.39	1.68	2.43	1.83	3.19	1.83
Games	2.30	1.24	2.38	1.19	2.18	1.49	2.30	1.40
Courses	1.58	0.81	1.65	0.77	1.21	0.57	1.43	0.76
Physical functioning	90.67	16.73	86.04	20.32	73.7	26.49	77.48	24.06

*Notes.* M = Mean; SD = Standard Deviation; DSST = Digit Symbol Substitution Test.

**Table 2.** Intercorrelations (Spearman) of the Study Variables for Middle-Aged adults and Older Adults (N = 5443).

	(1)	(2)	(3)	(4)	(5)	(6)	(7)	(8)	(9)	(10)	(11)	(12)	(13)
1. Cognitive speed (DSST)	—	−0.29***	0.06	0.25***	0.03	0.13***	0.28***	0.11***	0.23***	0.19***	0.18***	0.18***	0.20***
2. Age	−0.28***	—	−0.03	−0.11***	−0.04	−0.16***	−0.27***	−0.08**	0.02	−0.14***	−0.09**	−0.11***	−0.28***
3. Gender (female %)	0.18***	−0.02	—	−0.32***	0.03	−0.11***	−0.2***	0.07	0.11***	−0.03	0.04	−0.06	−0.15***
4. Education	0.22***	−0.01	−0.1***	—	−0.01	0.19***	0.40***	0.10***	0.04	0.27***	0.07	0.32***	0.16***
5. Employment (%)	0.24***	−0.45***	−0.07***	0.18***	—	0.01	−0.02	−0.03	−0.03	−0.01	0.01	0.00	0.01
6. Volunteering (%)	0.12***	−0.02	−0.04*	0.12***	0.05**	—	0.26***	0.06	0.00	0.18***	0.06	0.30***	0.13***
7. Computer use	0.26***	−0.12***	−0.08***	0.29***	0.18***	0.15***	—	0.09**	0.03	0.27***	0.08**	0.32***	0.21***
8. Visiting friends	0.11***	−0.09***	0.08***	0.06***	0.02	0.08***	0.09***	—	0.06	0.15***	0.2***	0.14***	0.10***
9. Brain games	0.08***	0.15***	0.14***	−0.06**	−0.15***	0.00	−0.03	0.04*	—	0.10***	0.24***	0.00	−0.01
10. Sports	0.19***	0.02	0.06***	0.20***	0.07***	0.12***	0.18***	0.18***	0.03	—	0.13***	0.26***	0.26***
11. Games	0.16***	−0.12***	0.08***	0.03*	0.04*	0.10***	0.06**	0.17***	0.17***	0.12***	—	0.10***	0.03
12. Courses	0.22***	−0.15***	0.04*	0.28***	0.19***	0.20***	0.22***	0.16***	−0.02	0.24***	0.10***	—	0.11***
13. Physical functioning	0.17***	−0.26***	−0.09***	0.16***	0.27***	0.03	0.08***	0.09***	−0.07***	0.17***	0.01	0.14***	—

*Notes.* Results for middle-aged adults are displayed on the lower triangle. Results for older adults are displayed on the upper triangle.

DSST = Digit Symbol Substitution Test; * *p* < .05; ** *p* < .01; *** *p* < .001.

To examine which variables might explain the observed improvements in cognitive speed over time, several linear regression analyses were conducted with increasing model complexity based on theoretical considerations, as depicted in Table 3. Regarding middle-aged adults, an effect of time period of *b* = 3.88 emerged, when controlling for age and gender (Table 1: Model 1). After additionally including education to the previous model, the effect size of time period decreased by 14% to *b* = 3.32 (Table 1: Model 2). Including working status and volunteering in addition to all previous variables further reduced the effect of time period by 12% to *b* = 2.84 (Table 1, Model 3). After additionally including participant’s routine daily activities, the effect size of time period strongly decreased by 50% to *b* = 0.89 (Table 1: Model 4). The last model included physical functioning in addition to all previous variables, and the effect size of time period increased again by 14% to *b* = 1.45 (Table 1: Model 5), suggesting that had physical functioning not strongly decreased over time even bigger improvements in cognitive speed would have been predicted to occur. Regarding older adults, an effect of time period of *b* = 2.98 emerged, when controlling for age and gender (Table 1: Model 1). After additionally including education to the previous model, the effect size of time period decreased by 43% to *b* = 1.69 (Table 1: Model 2). Including working status and volunteering in addition to all previous variables further reduced the effect of time period only slightly by 5% to *b* = 1.55 (Table 1, Model 3). After additionally including our set of routine activities, the effect size of time period again strongly decreased by 52% and completely disappeared to b = 0.00 (Table 1: Model 4). The last model included physical functioning in addition to all previous variables, and no more decreases in the effect of time period were found, with b = 0.00 (Table 1: Model 5).

**Table 3.** Linear Regression Analyses of Time Period and our Predictors predicting Cognitive Speed (N = 5443).

Middle-Aged Adults (N = 3330)															
	*Model 1*			*Model 2*			*Model 3*			*Model 4*			*Model 5*		
	*b*	*t*	*p*	*b*	*t*	*p*	*b*	*t*	*p*	*b*	*t*	*p*	*b*	*t*	*p*
Time period	3.88	8.60	<.001	3.32	7.56	<.001	2.84	6.45	<.001	0.89	1.87	.062	1.45	2.99	.003
Age	−0.51	−17.19	<.001	−0.50	−17.22	<.001	−0.41	−12.83	<.001	−0.38	−11.84	<.001	−0.37	−11.33	<.001
Gender (female %)	4.26	9.88	<.001	4.93	11.73	<.001	5.19	12.41	<.001	4.59	11.07	<.001	4.71	11.43	<.001
Education															
Low (Ref.)				1.00	—	—	1.00	—	—	1.00	—	—	1.00	—	—
Intermediate				6.22	6.45	<.001	5.60	5.84	<.001	4.24	4.52	<.001	3.75	4.02	<.001
Upper-intermediate				9.32	8.64	<.001	8.43	7.84	<.001	6.03	5.69	<.001	5.45	5.15	<.001
High				11.97	11.83	<.001	10.66	10.48	<.001	7.77	7.64	<.001	7.15	7.04	<.001
Working (%)							2.79	5.53	<.001	2.77	5.61	<.001	2.09	4.17	<.001
Volunteering (%)							2.67	5.63	<.001	1.68	3.58	<.001	1.64	3.53	<.001
Computer use										0.92	7.85	<.001	0.87	7.47	<.001
Visiting friends										0.10	0.44	.658	0.06	0.26	.794
Brain games										0.73	6.33	<.001	0.76	6.57	<.001
Sports										0.76	6.02	<.001	0.63	5.00	<.001
Games										0.60	3.51	<.001	0.63	3.71	<.001
Courses										0.68	2.50	.012	0.61	2.24	.025
Physical functioning													0.08	6.85	<.001
Older adults (*N* = 2113)															
	*Model 1*			*Model 2*			*Model 3*			*Model 4*			*Model 5*		
	*b*	*t*	*p*	*b*	*t*	*p*	*b*	*t*	*p*	*b*	*t*	*p*	*b*	*t*	*p*
Time period	2.98	5.47	<.001	1.69	3.13	.002	1.55	2.88	.004	0.00	0.01	.999	0.00	0.01	.993
Age	−0.71	−13.09	<.001	−0.62	−11.76	<.001	−0.6	−11.28	<.001	−0.52	−9.83	<.001	−0.46	−8.47	<.001
Gender (female %)	1.56	3.03	.002	3.68	6.93	<.001	3.76	7.07	<.001	3.15	5.99	<.001	3.54	6.72	<.001
Education															
Low (Ref.)				1.00	—	—	1.00	—	—	1.00	—	—	1.00	—	—
Intermediate				6.20	8.03	<.001	6.08	7.87	<.001	4.89	6.51	<.001	4.90	6.56	<.001
Upper-intermediate				7.33	7.40	<.001	7.15	7.20	<.001	5.21	5.35	<.001	5.19	5.37	<.001
High				11.15	12.13	<.001	10.87	11.75	<.001	7.97	8.42	<.001	7.91	8.42	<.001
Working (%)							1.75	0.40	.686	3.79	0.91	.363	3.69	0.89	.372
Volunteering (%)							1.59	2.53	.011	0.69	1.11	.268	0.59	0.95	.341
Computer use										0.79	5.67	<.001	0.76	5.54	<.001
Visiting friends										0.40	1.63	.104	0.31	1.26	.209
Brain games										1.12	9.27	<.001	1.11	9.26	<.001
Sports										0.22	1.59	.113	0.08	0.60	.545
Games										0.54	3.12	.002	0.58	3.37	<.001
Courses										0.54	1.44	.149	0.47	1.26	.207
Physical functioning													0.06	5.50	<.001

*Notes*. *b* = Regression coefficient; *t* = *t*-value; *p* = *p*-value.

## Discussion

We investigated potential explanatory factors for changes in cognitive functioning among middle-aged and older adults in the general population over historical time. We found that cognitive speed, a central dimension of cognitive functioning, improved in both middle-aged and older adults over time periods, from 2002 to 2014. Relative to their base values, increases in cognitive speed were slightly larger in older adults (increased mean cognitive speed scores of about 7% in 2014 relative to 2002) than in middle-aged adults (increased mean scores of about 10% in 2014 relative to 2002). In both age groups, these trends were partially explained by education, employment/volunteering status, and routine activities. However, physical functioning seemed mostly important only in explaining time trends in middle-aged adults (14% change in predicted time period differences in cognitive speed in middle-aged adults vs. 0% in older adults when controlling for physical functioning). Additionally, changes in education seemed more important to explain changes in older adults than in middle-aged adults (14% change in predicted time period differences in middle-aged adults vs. 43% in older adults when controlling for education). Thus, cognitive speed improved in both the middle-aged and older age group over time, and todays’ middle-aged and older adults exhibit higher degrees of cognitive functioning than middle-aged and older adults a decade ago. However, the relative descriptive importance of determinants for these increases differed between both age groups (Langa et al., 2008).

These results advance upon and are in line with those of previous studies. Longitudinal studies have reported increases of cognitive functioning over time (Trahan et al., 2014), but not much evidence is available on the relative strength of effects of potential factors contributing to these improvements. Most studies only focused on educational level (e.g., Weuve et al., 2018). Going beyond these previous studies, our findings add to this knowledge by considering multiple potentially important predictors that might explain improvements of cognitive speed, including employment status, volunteering status, routine activities, and health status. Several studies had already demonstrated that these factors might protect cognitive functioning in longitudinal studies (Kelly et al., 2017; Llewellyn et al., 2008; Menec, 2003; Walsemann & Ailshire, 2020). But, to our knowledge, this is the first study that examined whether these factors might also explain trends in cognitive functioning. We found that, indeed, education, occupational/volunteering status, and routine activities explained population-based improvements in cognitive speed over time. However, in contrast to previous studies, we found that the health status, as measured by physical functioning, explained trends in cognitive speed only among the middle-aged group. Interestingly, including physical functioning increased the trend coefficient of cognitive speed in middle-aged adults. This suggests that, had physical functioning not declined in middle-aged adults, even greater increases in cognitive speed would have been expected to occur. One potential explanation for the difference in the explanatory power of health status in our as compared to other studies might be that we included physical functioning as an indicator for health status, whereas some previous studies had included other health aspects like obesity (Hessel et al., 2018). Consequently, future studies are needed that examine the contribution of several different aspects of health to trends in cognitive functioning.

The results are also relevant from a theoretical perspective. Previous studies had hypothesized that an educational expansion was mostly responsible for improvements in cognitive functioning (Reimer & Pollak, 2010). We found this to be only partly true. Adding education to our regression models did indeed explain the observed increases strongly in older adults, but only to a smaller degree in middle-aged adults. This suggests that further explanatory factors are needed, especially those focusing on changing behavioral patterns and health over time, as suggested by our results.

Additionally, the results are important from a practical perspective. Increases in cognitive functioning that could be explained by education have mostly been obtained in older cohorts. In middle-aged cohorts, the improvements in cognitive speed due to education can only be described as small. This suggests that education should not be expected to automatically lead to further increases in cognitive functioning in future younger cohorts. Instead, it seems that behavioral patterns of regular activities are much more important in explaining trends in middle-aged participants and potential future cohorts. These routine activities should thus be emphasized in future research and intervention studies. Further, it seems worrying that physical functioning has decreased over time in middle-aged adults—a finding that has also been reported in some previous studies—,pointing to a possible expansion of cognitive morbidity in the future (Beller et al., 2019, 2020, 2021; Beller & Epping, 2020; Gruenberg, 1977; Kramer, 1980). Future studies should examine the reasons for this decline in physical functioning among middle-aged adults.

Despite the novelty of our findings and the strengths of this study, a number of limitations must be acknowledged. First, the current study focused only on a single indicator of cognitive functioning, cognitive speed as measured by the Digit Symbol Substitution Test (Jaeger, 2018). Results might differ when other, more specific aspects of cognitive functioning like short- and long-term memory were to be analyzed, which should be done by future studies. However, it must also be noted that the Digit Symbol Substitution Test represents one of the most widely used and acknowledged cognitive tests and it has been shown to correlate strongly with a broad range of higher cognitive functions (Salthouse, 1996). Second, this study only analyzed data from middle-aged and older adults of one country, Germany. Although findings regarding changes in cognitive functioning have generally been consistent across countries, some differences also emerged in previous studies. Thus, our analyses should be replicated in other countries. In a similar vein, other complementary methodological approaches like the analysis of trajectories in cognitive functioning across cohorts could be used. Third, although the German Aging Survey has been widely used by scientists as a data source to study how people age in Germany, we cannot rule out that selectivities in the sample might have led to biased trends, such as an underestimation of the effect of education on changes in cognitive speed (Klaus et al., 2017). Fourth, our explanatory factors focused on variables related to socio-demography and routine activities. Future studies should include further predictors that have been linked to cognitive performance, such as loneliness, sleep patterns, nutrition, and working activities (Andel et al., 2015; Beller & Wagner, 2018; Gehlich et al., 2019a, 2019b; Virta et al., 2013). Last, our statistical analyses are only based on observational regression methods, and thus causality cannot be ascertained. To attenuate this problem, more evidence regarding the explanations of health trends is needed.

## Appendix

**AppendixTable A1.** Linear Regression Analyses of Time Period and our Predictors predicting Cognitive Speed using an Imputed Datset (*N* = 7136).

Middle-Aged Adults (*N* = 4215)															
	*Model 1*			*Model 2*			*Model 3*			*Model 4*			*Model 5*		
	*b*	*t*	*p*	*b*	*t*	*p*	*b*	*t*	*p*	*b*	*t*	*p*	*b*	*t*	*p*
Time period	3.85	10.73	<.001	3.21	9.19	<.001	2.60	7.45	<.001	0.66	1.74	.081	1.21	3.17	.002
Age	−0.51	−21.21	<.001	−0.49	−21.15	<.001	−0.40	−15.52	<.001	−0.36	−14.05	<.001	−0.34	−13.51	<.001
Gender (female %)	3.88	11.07	<.001	4.61	13.52	<.001	4.89	14.46	<.001	4.39	13.14	<.001	4.50	13.56	<.001
Education															
Low (Ref.)				1.00	—	—	1.00	—	—	1.00	—	—	1.00	—	—
Intermediate				5.99	7.95	<.001	5.34	7.16	<.001	3.80	5.24	<.001	3.43	4.76	<.001
Upper-intermediate				9.12	10.74	<.001	8.12	9.62	<.001	5.48	6.60	<.001	5.03	6.09	<.001
High				11.59	14.56	<.001	10.18	12.77	<.001	7.12	8.94	<.001	6.60	8.32	<.001
Working (%)							3.14	7.76	<.001	3.03	7.67	<.001	2.35	5.87	<.001
Volunteering (%)							2.67	6.87	<.001	1.65	4.30	<.001	1.59	4.20	<.001
Computer use										0.95	10.08	<.001	0.90	9.62	<.001
Visiting friends										0.09	0.49	.624	0.04	0.23	.819
Brain games										0.69	7.44	<.001	0.72	7.77	<.001
Sports										0.72	7.11	<.001	0.61	5.98	<.001
Games										0.64	4.63	<.001	0.65	4.80	<.001
Courses										0.68	3.10	.002	0.61	2.82	.005
Physical functioning													0.07	8.24	<.001
Older adults (*N* = 2921)															
	*Model 1*			*Model 2*			*Model 3*			*Model 4*			*Model 5*		
	*b*	*t*	*p*	*b*	*t*	*p*	*b*	*t*	*p*	*b*	*t*	*p*	*b*	*t*	*p*
Time period	3.23	8.09	<.001	1.93	4.94	<.001	1.78	4.53	<.001	0.07	0.18	.857	0.04	0.11	.915
Age	−0.69	−17.48	<.001	−0.62	−16.22	<.001	−0.60	−15.57	<.001	−0.51	−13.5	<.001	−0.44	−11.49	<.001
Gender (female %)	1.19	3.08	0.002	3.20	8.10	<.001	3.28	8.31	<.001	2.65	6.86	<.001	3.03	7.85	<.001
Education															
Low (Ref.)				1.00	—	—	1.00	—	—	1.00	—	—	1.00	—	—
Intermediate				6.02	10.72	<.001	5.87	10.46	<.001	4.66	8.61	<.001	4.58	8.54	<.001
Upper-intermediate				7.05	9.49	<.001	6.85	9.21	<.001	4.91	6.79	<.001	4.81	6.71	<.001
High				10.75	15.87	<.001	10.43	15.32	<.001	7.45	10.75	<.001	7.29	10.61	<.001
Working (%)							1.75	0.52	.601	4.01	1.26	.209	3.57	1.13	.259
Volunteering (%)							1.82	3.74	<.001	0.86	1.78	.075	0.73	1.53	.125
Computer use										0.77	7.29	<.001	0.76	7.25	<.001
Visiting friends										0.38	2.15	.032	0.26	1.49	.136
Brain games										1.07	11.96	<.001	1.06	11.89	<.001
Sports										0.26	2.52	.012	0.11	1.07	.287
Games										0.53	4.12	<.001	0.54	4.26	<.001
Courses										0.56	1.98	.047	0.46	1.65	.099
Physical functioning													0.05	7.33	<.001

*Notes*. *b* = Regression coefficient; *t* = *t*-value; *p* = *p*-value.

**Table A2.** Linear Regression Analyses of Time Period and our Predictors predicting Cognitive Speed with Weights (*N* = 5443).

Middle-Aged Adults (*N* = 3330)															
	*Model 1*			*Model 2*			*Model 3*			*Model 4*			*Model 5*		
	*b*	*t*	*p*	*b*	*t*	*p*	*b*	*t*	*p*	*b*	*t*	*p*	*b*	*t*	*p*
Time period	4.34	9.51	<.001	3.81	8.53	<.001	3.32	7.49	<.001	1.41	2.87	.004	1.87	3.80	<.001
Age	−0.51	−17.44	<.001	−0.49	−17.25	<.001	−0.41	−13.13	<.001	−0.38	−12.17	<.001	−0.36	−11.44	<.001
Gender (female %)	4.32	10.11	<.001	4.96	11.81	<.001	5.28	12.67	<.001	4.75	11.45	<.001	4.87	11.8	<.001
Education															
Low (Ref.)				1.00	—	—	1.00	—	—	1.00	—	—	1.00	—	—
Intermediate				5.59	5.92	<.001	4.56	4.84	<.001	3.28	3.55	<.001	2.80	3.04	.002
Upper-intermediate				8.19	7.67	<.001	6.87	6.45	<.001	4.82	4.56	<.001	4.26	4.05	<.001
High				11.04	11.11	<.001	9.37	9.38	<.001	6.96	6.93	<.001	6.35	6.34	<.001
Working (%)							3.53	6.68	<.001	3.39	6.54	<.001	2.70	5.13	<.001
Volunteering (%)							2.86	6.18	<.001	2.03	4.42	<.001	1.99	4.36	<.001
Computer use										0.88	7.11	<.001	0.84	6.87	<.001
Visiting friends										−0.11	−0.48	.629	−0.12	−0.55	.580
Brain games										0.74	6.10	<.001	0.76	6.37	<.001
Sports										0.68	5.32	<.001	0.56	4.37	<.001
Games										0.66	3.80	<.001	0.67	3.91	<.001
Courses										0.62	2.29	.022	0.55	2.04	.041
Physical functioning													0.08	6.47	<.001
Older adults (*N* = 2113)															
	*Model 1*			*Model 2*			*Model 3*			*Model 4*			*Model 5*		
	*b*	*t*	*p*	*b*	*t*	*p*	*b*	*t*	*p*	*b*	*t*	*p*	*b*	*t*	*p*
Time period	3.25	5.89	<.001	2.08	3.83	<.001	1.89	3.46	.001	0.39	0.70	.486	0.41	0.73	.464
Age	−0.70	−13.57	<.001	−0.60	−11.96	<.001	−0.58	−11.3	<.001	−0.50	−9.81	<.001	−0.44	−8.34	<.001
Gender (female %)	1.50	2.93	.003	3.49	6.62	<.001	3.60	6.81	<.001	3.04	5.78	<.001	3.43	6.51	<.001
Education															
Low (Ref.)				1.00	—	—	1.00	—	—	1.00	—	—	1.00	—	—
Intermediate				5.87	8.13	<.001	5.70	7.88	<.001	4.56	6.48	<.001	4.56	6.52	<.001
Upper-intermediate				6.96	7.04	<.001	6.68	6.74	<.001	4.81	4.93	<.001	4.86	5.02	<.001
High				10.62	11.93	<.001	10.26	11.47	<.001	7.65	8.32	<.001	7.58	8.29	<.001
Working (%)							1.93	0.55	.582	3.72	1.10	.271	3.65	1.09	.277
Volunteering (%)							2.00	3.29	.001	1.10	1.80	.072	0.97	1.61	.107
Computer use										0.74	5.35	<.001	0.73	5.30	<.001
Visiting friends										0.41	1.68	.094	0.35	1.43	.153
Brain games										1.08	8.94	<.001	1.06	8.8	<.001
Sports										0.22	1.61	.107	0.08	0.56	.579
Games										0.62	3.57	<.001	0.67	3.90	<.001
Courses										0.49	1.37	.171	0.41	1.14	.254
Physical functioning													0.06	5.55	<.001

*Notes*. *b* = Regression coefficient; *t* = *t*-value; *p* = *p*-value.

## References

[bibr1-08982643211065571] Andel R. Silverstein M. Kåreholt I. (2015). The role of midlife occupational complexity and leisure activity in late-life cognition. The Journals of Gerontology Series B: Psychological Sciences and Social Sciences, 70(2), 314–321. 10.1093/geronb/gbu110 25190210

[bibr2-08982643211065571] Beller J. Bauersachs J. Schäfer A. Schwettmann L. Heier M. Peters A. Meisinger C. Geyer S. (2020). Diverging trends in age at first myocardial infarction: Evidence from two German Population-based studies. Scientific Reports, 10(1), 9610. 10.1038/s41598-020-66291-4 32541657 PMC7296035

[bibr3-08982643211065571] Beller J. Epping J. (2020). Disability trends in Europe by age-period-cohort analysis: Increasing disability in younger cohorts. Disability and Health Journal, 14(1), 100948. 10.1016/j.dhjo.2020.100948 32690322

[bibr4-08982643211065571] Beller J. Miething A. Regidor E. Lostao L. Epping J. Geyer S. (2019). Trends in grip strength: Age, period, and cohort effects on grip strength in older adults from Germany, Sweden, and Spain. SSM Population Health, 9, 100456. 10.1016/j.ssmph.2019.100456 31453311 PMC6700453

[bibr5-08982643211065571] Beller J. Regidor E. Lostao L. Miething A. Kröger C. Safieddine B. Tetzlaff F. Sperlich S. Geyer S. (2021). Decline of depressive symptoms in Europe: Differential trends across the lifespan. Social Psychiatry and Psychiatric Epidemiology, 56(7), 1249–1262. 10.1007/s00127-020-01979-6 33180149 PMC8225536

[bibr6-08982643211065571] Beller J. Wagner A. (2018). Disentangling loneliness: Differential effects of subjective loneliness, network quality, network size, and living alone on physical, mental, and cognitive health. Journal of Aging and Health, 30(4), 521–539. 10.1177/0898264316685843 28553795

[bibr7-08982643211065571] Bullinger M. (1995). German translation and psychometric testing of the SF-36 Health Survey: Preliminary results from the IQOLA project. Social Science & Medicine, 41(10), 1359–1366. 10.1016/0277-9536(95)00115-N 8560303

[bibr8-08982643211065571] Fries J. F. (1980). Aging, natural death, and the compression of morbidity. New England Journal of Medicine, 303(3), 130–135. 10.1056/NEJM198007173030304 7383070

[bibr9-08982643211065571] Fries J. F. Bruce B. Chakravarty E. (2011). Compression of morbidity 1980–2011: A focused review of paradigms and progress. Journal of Aging Research, 2011, 261702. 10.4061/2011/261702 21876805 PMC3163136

[bibr10-08982643211065571] Gehlich K. H. Beller J. Lange-Asschenfeldt B. Köcher W. Meinke M. C. Lademann J. (2019a). Consumption of fruits and vegetables: Improved physical health, mental health, physical functioning and cognitive health in older adults from 11 European countries. Aging & Mental Health, 10.1080/13607863.2019.1571011 30729805

[bibr11-08982643211065571] Gehlich K. H. Beller J. Lange-Asschenfeldt B. Köcher W. Meinke M. C. Lademann J. (2019b). Fruit and vegetable consumption is associated with improved mental and cognitive health in older adults from non-Western developing countries. Public Health Nutrition, 22(4), 689–696. 10.1017/S1368980018002525 30295221 PMC10260677

[bibr12-08982643211065571] Grasshoff J. Beller J. Kuhlmann B. G. Geyer S. (2021). Increasingly capable at the ripe old age? Cognitive abilities from 2004 to 2013 in Germany, Spain, and Sweden. PLoS One, 16(7), e0254038. 10.1371/journal.pone.0254038 34197534 PMC8248634

[bibr13-08982643211065571] Gruenberg E. M. (1977). The failures of success. The Milbank Memorial Fund QuarterlyHealth and Society, 55(1), 3. 10.2307/3349592 141009

[bibr14-08982643211065571] Hale J. M. Schneider D. C. Gampe J. Mehta N. K. Myrskylä M. (2020). Trends in the risk of cognitive impairment in the United States, 1996–2014. Epidemiology, 31(5), 745–754. 10.1097/EDE.0000000000001219 32740472 PMC7386871

[bibr15-08982643211065571] Henchoz Y. Büla C. von Gunten A. Blanco J. M. Seematter-Bagnoud L. Démonet J.-F. Waeber G. Nanchen D. Santos-Eggimann B. (2020). Trends in physical and cognitive performance among community-dwelling older adults in Switzerland. The Journals of Gerontology: Series A, 75(12), 2347–2353. 10.1093/gerona/glaa008 31942995

[bibr16-08982643211065571] Hessel P. Kinge J. M. Skirbekk V. Staudinger U. M. (2018). Trends and determinants of the Flynn effect in cognitive functioning among older individuals in 10 European countries. Journal of Epidemiology and Community Health, 72(5), 383–389. 10.1136/jech-2017-209979 29440306

[bibr17-08982643211065571] Hoyer W. J. Stawski R. S. Wasylyshyn C. Verhaeghen P. (2004). Adult age and digit symbol substitution performance: A meta-analysis. Psychology and Aging, 19(1), 211–214. 10.1037/0882-7974.19.1.211 15065945

[bibr18-08982643211065571] Jaeger J. (2018). Digit symbol substitution test: The case for sensitivity over specificity in neuropsychological testing. Journal of Clinical Psychopharmacology, 38(5), 513–519. 10.1097/JCP.0000000000000941 30124583 PMC6291255

[bibr19-08982643211065571] Kelly M. E. Duff H. Kelly S. McHugh Power J. E. Brennan S. Lawlor B. A. Loughrey D. G. (2017). The impact of social activities, social networks, social support and social relationships on the cognitive functioning of healthy older adults: A systematic review. Systematic Reviews, 6(1), 259. 10.1186/s13643-017-0632-2 29258596 PMC5735742

[bibr20-08982643211065571] Klaus D. Engstler H. Mahne K. Wolff J. K. Simonson J. Wurm S. Tesch-Römer C. (2017). Cohort profile: The German Ageing Survey (DEAS). International Journal of Epidemiology, 46(4), 1105–1105g. 10.1093/ije/dyw326 28180273 PMC5837219

[bibr21-08982643211065571] Kramer M. (1980). The rising pandemic of mental disorders and associated chronic diseases and disabilities. Acta Psychiatrica Scandinavica, 62(S285), 382–397. 10.1111/j.1600-0447.1980.tb07714.x7468297

[bibr22-08982643211065571] Langa K. M. Larson E. B. Karlawish J. H. Cutler D. M. Kabeto M. U. Kim S. Y. Rosen A. B. (2008). Trends in the prevalence and mortality of cognitive impairment in the United States: Is there evidence of a compression of cognitive morbidity? Alzheimer’s & Dementia, 4(2), 134–144. 10.1016/j.jalz.2008.01.001 PMC2390845 18631957

[bibr23-08982643211065571] Llewellyn D. J. Lang I. A. Langa K. M. Huppert F. A. (2008). Cognitive function and psychological well-being: Findings from a population-based cohort. Age and Ageing, 37(6), 685–689. 10.1093/ageing/afn194 18852289 PMC2720691

[bibr24-08982643211065571] MacKinnon D. P. (2000). Equivalence of the mediation, confounding and suppression effect. Prevention Science, 1(4), 173–181. 10.1023/A:1026595011371 11523746 PMC2819361

[bibr25-08982643211065571] Mahne K. Wolff J. Tesch-Römer C. (2020). Scientific Use File German Ageing Survey (SUF DEAS) 2014, Version 3.0Scientific Use File Deutscher Alterssurvey (SUF DEAS) 2014 Version 3.0 (3.0) [Data set]. DZA The German Centre of Gerontology. 10.5156/DEAS.2014.M.005

[bibr26-08982643211065571] Menec V. H. (2003). The relation between everyday activities and successful aging: A 6-Year Longitudinal Study. The Journals of Gerontology Series B: Psychological Sciences and Social Sciences, 58(2), S74–S82. 10.1093/geronb/58.2.S74 12646596

[bibr27-08982643211065571] Motel-Klingebiel A. Tesch-Römer C. Wurm S. (2016). Scientific Use File Deutscher Alterssurvey (SUF DEAS) 2008 Version 3.0 [Data set]. DZA The German Centre of Gerontology. 10.5156/DEAS.2008.M.003

[bibr28-08982643211065571] Reimer D. Pollak R. (2010). Educational expansion and its consequences for Vertical and Horizontal inequalities in access to higher education in West Germany. European Sociological Review, 26(4), 415–430. 10.1093/esr/jcp029

[bibr29-08982643211065571] Salthouse T. A. (1996). The processing-speed theory of adult age differences in cognition. Psychological Review, 103(3), 403–428. 10.1037/0033-295X.103.3.403 8759042

[bibr30-08982643211065571] Stekhoven D. J. Buhlmann P. (2012). MissForest—Non-parametric missing value imputation for mixed-type data. Bioinformatics, 28(1), 112–118. 10.1093/bioinformatics/btr597 22039212

[bibr31-08982643211065571] Trahan L. H. Stuebing K. K. Fletcher J. M. Hiscock M. (2014). The Flynn effect: A meta-analysis. Psychological Bulletin, 140(5), 1332–1360. 10.1037/a0037173 24979188 PMC4152423

[bibr32-08982643211065571] Virta J. J. Heikkilä K. Perola M. Koskenvuo M. Räihä I. Rinne J. O. Kaprio J. (2013). Midlife sleep characteristics associated with late life cognitive function. Sleep, 36(10), 1533–1541. 10.5665/sleep.3052 24082313 PMC3773203

[bibr33-08982643211065571] Walsemann K. M. Ailshire J. A. (2020). Early educational experiences and trajectories of cognitive functioning among US adults in midlife and later. American Journal of Epidemiology, 189(5), 403–411. 10.1093/aje/kwz276 31907547 PMC7443204

[bibr34-08982643211065571] Weuve J. Rajan K. B. Barnes L. L. Wilson R. S. Evans D. A. (2018). Secular trends in cognitive performance in older Black and White U.S. Adults, 1993–2012: Findings from the chicago health and aging project. The Journals of Gerontology: Series B, 73(suppl_1), S73–S81. 10.1093/geronb/gbx167 PMC6019012 29669103

